# Infective Endocarditis Secondary to Needle Embolization to the Heart: A Case Report

**DOI:** 10.7759/cureus.61459

**Published:** 2024-05-31

**Authors:** Tony Elias, Kyrillos Girgis, Maziyar Daneshvar, Howard Weinberg, David M Barsoum, Robert Malak, Veyola Rezkalla, Rafail Beshai

**Affiliations:** 1 Internal Medicine, Rowan-Virtua School of Osteopathic Medicine, Stratford, USA; 2 Internal Medicine, Newark Beth Israel Medical Center, Newark, USA; 3 Cardiology, Virtua Health, Camden, USA; 4 Cardiovascular Disease, Virtua Health, Camden, USA; 5 Internal Medicine, Jefferson Health, Stratford, USA

**Keywords:** bacteremia, intravenous drug use, needle embolization, rare presentation, infective endocarditis

## Abstract

This case report explores the rare occurrence of a needle embolism in the heart among individuals with intravenous drug use (IVDU). The intricate symptomatology, ranging from overt chest pain to asymptomatic cases, poses diagnostic challenges and may lead to underrecognition. Healthcare professionals must navigate varied presentations, emphasizing the need for a nuanced diagnostic approach. The interplay of needle embolisms with infective endocarditis and sepsis adds complexity, requiring a comprehensive understanding. Ongoing education and training are crucial for healthcare professionals to address the evolving challenges of needle embolism management within the broader context of infective endocarditis and sepsis. Our patient is a 31-year-old female with a history of IVDU who presented with heart palpitations and shortness of breath. A CT scan revealed lung lesions and a needle in the right ventricle. The patient was admitted for methicillin-sensitive *Staphylococcus aureus *(MSSA) bacteremia, where she underwent video-assisted thoracoscopic surgery (VATS) involving empyemectomy and wedge resection of the right-middle and lower lobes. However, it was deemed very risky to remove the needle from the right ventricle. Despite extensive discussion and patient education, she left the rehabilitation center without follow-up, highlighting the challenges of managing IV drug-related complications. In conclusion, heightened awareness and a proactive approach are crucial in managing rare complications such as needle embolisms in IVDU patients. This case underscores the significance of staying informed to improve patient care and outcomes amid evolving healthcare practices.

## Introduction

The escalating prevalence of intravenous drug use (IVDU) in recent years has brought attention to the consequential rise in needle embolisms in the heart, an infrequent yet clinically significant occurrence [1]. This cardiac complication arises from the inadvertent introduction of foreign bodies, such as needles, into the circulatory system. The symptoms associated with needle embolisms in the heart present a diverse spectrum, ranging from conspicuous manifestations such as chest pain and dyspnea to the subtler and intriguing possibility of asymptomatic presentation [2,3]. The multifaceted nature of the symptomology, compounded by instances of asymptomatic cases, intensifies the intricacy of diagnosing needle embolisms in the heart, contributing to potential underrecognition within clinical settings. In exploring the complexity of this condition, healthcare professionals must navigate the challenges of varied symptom presentations. Chest pain and dyspnea, while more overt, may be accompanied by subtle or absent symptoms, adding layers of difficulty to an accurate and timely diagnosis [2]. The rarity of needle embolisms necessitates heightened awareness among healthcare professionals, emphasizing the need for a nuanced diagnostic approach. Swift and accurate medical responses become pivotal in optimizing patient outcomes, underscoring the critical importance of timely diagnosis and intervention.

To appreciate the gravity of needle embolisms in the heart, it is essential to delve into the broader context of infective endocarditis (IE) and sepsis, conditions often associated with intravenous drug abuse. IE is a serious and potentially life-threatening condition characterized by the inflammation of the endocardium, typically involving the heart valves [4]. In individuals with IVDU, the risk of developing IE is notably heightened due to the introduction of infectious agents through contaminated needles. The microbial invasion of the heart valves can lead to the formation of vegetations, causing valvular dysfunction and the potential for emboli formation [5,6].

Recent research by Zaree et al. [7] emphasizes the ongoing imperative for education and training within the medical community to enhance proficiency in identifying and addressing intricate cardiac issues. Their findings underscore the need for healthcare professionals to stay abreast of evolving healthcare practices, recognizing the dynamic nature of the field. Continuous efforts aimed at expanding knowledge and refining diagnostic strategies are imperative, ensuring ongoing improvement in the recognition and management of needle embolisms in the heart, especially within the broader context of IE and sepsis. The interplay of these conditions requires a comprehensive approach, reflecting the evolving landscape of healthcare and the increasing challenges posed by intravenous drug abuse.

## Case presentation

**Figure 1:**
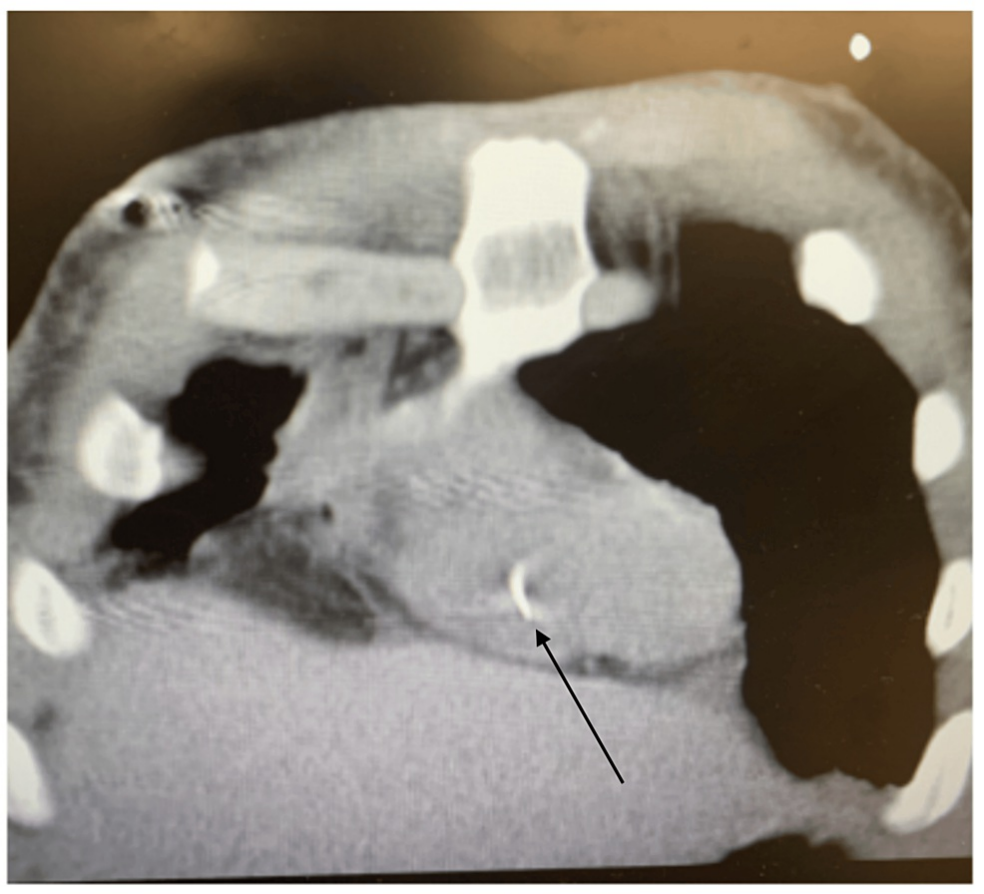
Chest CT coronal view showing a radio-opaque foreign object in the right ventricle (black arrow).

**Figure 2:**
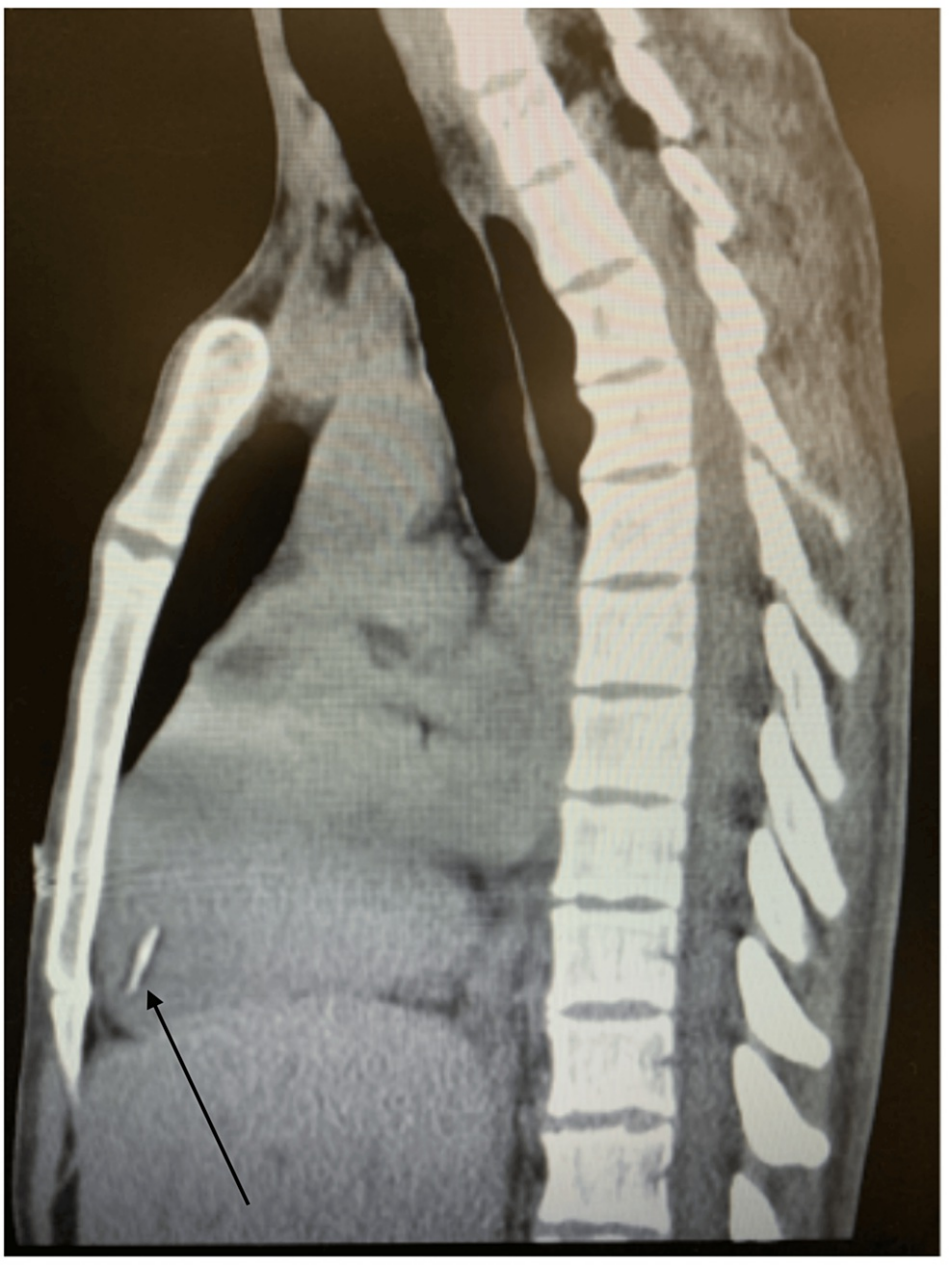
Chest CT sagittal view showing a radio-opaque foreign object in the right ventricle (black arrow).

A 31-year-old female with a history of IVDU presented to the emergency room reporting heart palpitations and a peculiar sensation in her chest, accompanied by shortness of breath. Vitals showed a heart rate of 140 beats per minute (BPM), blood pressure of 110/ 70 mmHg, respiratory rate of 18 breaths per minute, temperature of 100.8 °F, and oxygen saturation of 94%. An electrocardiogram (EKG) revealed only sinus tachycardia. Labs showed elevated white blood cells (WBCs) of 30 × 109/L, C-reactive protein of 10 mg/dL, erythrocyte sedimentation rate (ESR) of 130 mm/hour, procalcitonin of 2.1 ng/mL, and high sensitivity troponin of 210 ng/L. During the evaluation, she disclosed recent IV heroin use, pinpointing her last instance just two days prior to seeking medical attention. A computed tomography (CT) scan of the chest unveiled bilateral lung complex cavitary lesions, raising concerns about septic emboli, along with the identification of a linear foreign object in the right ventricle (Figures 1, 2).

Broad-spectrum antibiotics of vancomycin and Zosyn were initiated. Subsequently, a transesophageal echocardiogram was conducted, revealing a needle fragment embedded in the right ventricular (RV) apex, extending into the septum. Blood cultures confirmed methicillin-sensitive *Staphylococcus aureus* (MSSA) bacteremia, and antibiotics were deescalated to Ancef.

Consultation with the cardiothoracic surgery (CTS) department led to the patient undergoing right video-assisted thoracoscopic surgery (VATS) involving empyemectomy and wedge resection of the right-middle and lower lobes. However, CTS expressed concerns about the potential destructiveness of searching for the needle in the operating room, considering the current normal structure of the right ventricle. After extensive deliberation, it was determined that surgical intervention would only be necessary if the needle embolized to the pulmonary artery branches, caused local perforation due to inflammation, or became infected, forming an abscess. Following this decision, the patient was discharged to an inpatient drug rehabilitation center, where she received an additional three weeks of intravenous antibiotics with Ancef. Unfortunately, she did not follow up with her primary care office appointments subsequent to her stay in the rehabilitation center.

## Discussion

This extraordinary case study unveils a clinical scenario where a patient, initially presenting with heart palpitations, was ultimately diagnosed with a needle embolism in the heart. This unique occurrence underscores the diverse and unexpected manifestations associated with IVDU, emphasizing the need for heightened vigilance among healthcare professionals in diagnosing and managing atypical presentations.

IVDU has long been recognized as a significant risk factor for IE, a severe and potentially life-threatening condition characterized by inflammation of the endocardium, often involving the heart valves [4,8]. The microbial invasion of the heart valves can result in the formation of vegetations, causing valvular dysfunction and emboli that may travel to various parts of the body, including the lungs, brain, and other vital organs [9]. The patient's initial presentation with heart palpitations prompted considerations of IE, a condition known for its diverse clinical manifestations.

The emergence of sepsis becomes a critical concern in the context of IE, as the microbial invasion can lead to the systemic dissemination of infection. The inflammatory response triggered by the infection can result in a dysregulated immune response, culminating in sepsis, a life-threatening condition characterized by organ dysfunction [6].

While the needle embolism represented a rare and unusual complication, as only five cases were found in our literature review, it became evident that healthcare professionals should remain vigilant about potential complications arising from IVDU. The multifaceted symptomology associated with IE and the possibility of asymptomatic needle embolisms contribute to the complexity of diagnosis, emphasizing the importance of considering rare etiologies in patients with a history of IVDU. This case report not only expands our understanding of the varied presentations of IVDU-related complications but also serves as a reminder of the need for comprehensive and interdisciplinary management in such complex cases. This unique insight contributes to a broader perspective on the challenges and considerations in diagnosing and managing uncommon cardiac complications, ultimately benefiting healthcare professionals, researchers, and policymakers aiming to enhance patient care and outcomes in similar clinical scenarios.

## Conclusions

As we contemplate the implications of this case, it becomes apparent that further research is warranted to explore the prevalence of needle embolisms in individuals with IVDU and to establish standardized guidelines for the diagnosis and management of such cases.

This case not only adds a unique dimension to the existing knowledge base but also emphasizes the need for ongoing education and training within the medical community to enhance proficiency in identifying and addressing intricate cardiac issues. The dynamic nature of healthcare requires continuous efforts to expand knowledge and refine diagnostic strategies, ensuring improved recognition and management of needle embolisms in the context of IE.
